# Genetic structure of *Cercospora beticola* populations on *Beta vulgaris* in New York and Hawaii

**DOI:** 10.1038/s41598-017-01929-4

**Published:** 2017-05-11

**Authors:** Niloofar Vaghefi, Scot C. Nelson, Julie R. Kikkert, Sarah J. Pethybridge

**Affiliations:** 1 School of Integrative Plant Science, Plant Pathology & Plant-Microbe Biology Section, Cornell University, Geneva, NY 14456 USA; 2 College of Tropical Agriculture and Human Resources, Department of Tropical Plant and Soil Sciences, University of Hawaii at Manoa, Honolulu, HI 96822 USA; 3 Cornell Cooperative Extension, Canandaigua, NY 14424 USA

## Abstract

Cercospora leaf spot (CLS), caused by *Cercospora beticola*, is a major disease of *Beta vulgaris* worldwide. No sexual stage is known for *C. beticola* but in its asexual form it overwinters on infected plant debris as pseudostromata, and travels short distances by rain splash-dispersed conidiospores. *Cercospora beticola* infects a broad range of host species and may be seedborne. The relative contribution of these inoculum sources to CLS epidemics on table beet is not well understood. Pathogen isolates collected from table beet, Swiss chard and common lambsquarters in mixed-cropping farms and monoculture fields in New York and Hawaii, USA, were genotyped (*n* = 600) using 12 microsatellite markers. All isolates from CLS symptoms on lambsquarters were identified as *C. chenopodii*. Sympatric populations of *C. beticola* derived from Swiss chard and table beet were not genetically differentiated. Results suggested that local (within field) inoculum sources may be responsible for the initiation of CLS epidemics in mixed-cropping farms, whereas external sources of inoculum may be contributing to CLS epidemics in the monoculture fields in New York. New multiplex PCR assays were developed for mating-type determination for *C. beticola*. Implications of these findings for disease management are discussed.

## Introduction

Population genetic studies of plant pathogens using selectively neutral genetic markers have made significant contributions to plant disease management in the past few decades^1, 2^. These studies apply knowledge of the genetic composition and patterns of genetic diversity in pathogen populations to test hypotheses regarding their biology and plant disease epidemiology^3–5^. Population genetics has been used to make inferences about the origin^6–9^, sources of inoculum^10, 11^, routes of introduction^12, 13^, migration pathways^7, 14–17^ and reproductive biology^18, 19^ of plant pathogens. Knowledge of the reproductive modes of plant pathogens is critical to disease management, due to its significant impacts on the evolutionary trajectory and epidemiology of pathogen populations. Recombination as a result of sexual reproduction is an influential evolutionary force that generates potentially adaptive genotypic diversity, purges deleterious mutations, and produces sexual propagules with enhanced survival and dispersal^20^. In the absence of a known sexual stage for a plant pathogen, population genetic studies can provide indirect evidence to confirm the asexuality of pathogen populations^21–23^, or reveal historical or potentially cryptic sex in presumably asexual fungi^18, 19, 24^. In pathogen populations with frequent sexual reproduction, high genotypic diversity, random association of selectively neutral genetic loci and equal distribution of the opposite mating-types (for heterothallic fungi) are expected. Alternatively, a strictly asexual population is characterized by low genotypic diversity, lack of recombinant genotypes, widespread occurrence of a few clonal lineages, non-random association of unlinked loci, and skewed distribution of alternate mating-types in the population^20^.

The presence of a sexual form remains unknown for the globally and economically important plant pathogen, *Cercospora beticola*, the cause of Cercospora leaf spot (CLS) of *Beta vulgaris* L. (sugar beet, table beet and Swiss chard)^25^. Despite the lack of a known sexual form, *C. beticola* is considered to be a heterothallic, ascomycete fungus due to the discovery of two alternate mating-type genes (*MAT1-1-1* and *MAT1-2-1*) at equal frequency in parts of Europe^26, 27^. Population genetic studies have also suggested the potential presence of a sexual cycle for *C. beticola* in sugar beet production areas in North Dakota^28^. In contrast, no evidence for sexual reproduction has been found in other parts of the USA^29^ or the Middle East^30^.

In New York, CLS epidemics impose significant losses to both the fresh market and processing table beet (*Beta vulgaris* ssp. *vulgaris*) industries^25^. Symptoms include light brown to grey leaf spots, usually with a red to purple margin in red beet varieties, which expand into necrotic lesions that result in premature defoliation^31^. Conidiospores are mainly disseminated through water splash over short distances, resulting in multiple infection cycles within a season and polycyclic epidemics^25^.

Infested plant debris is considered to be a major source of inoculum initiating CLS epidemics^32–34^ as *C. beticola* in the form of mycelia and pseudostromata may persist for a maximum of 22 months^35^ to three years^36^ depending on climatic conditions. In small-scale, mixed-cropping farms in New York, table beet and Swiss chard (*B. vulgaris* ssp. *cicla*) are often grown in close proximity along with other vegetables, on an annual basis. It is, therefore, plausible that infested plant debris from previous years may be a major source of inoculum contributing to the annual CLS epidemics on susceptible hosts in these farms. In contrast, large-scale monoculture table beet fields in New York involve rotations with non-host crops in the same field for a recommended minimum duration of three years, which aims to reduce the persistence of inoculum in soil and on infested plant debris. Nevertheless, CLS epidemics are prevalent and result in substantial losses in these fields, even with rotations lasting up to five years^37^, and even in the fields that have never grown table beet (George Abawi, *personal communication*). The major sources of initial inoculum for CLS epidemics in monoculture table beet fields in New York are therefore largely unknown.

Understanding the relative contribution of inoculum sources to CLS epidemics is essential for the design of effective management strategies. If *C. beticola* populations in New York are capable of sexual reproduction, overwintering sexual structures may serve as primary inoculum for newly established table beet fields within several kilometres. *Cercospora beticola* is also known to infect a broad range of plants from various families^32, 38^. Therefore, infected weeds (especially from *Amaranthaceae*) are believed to serve as green bridges for *C. beticola* inocula during crop rotations. For example, common lambsquarters (*Chenopodium album*) is a susceptible weed species that is frequently found in New York table beet fields and often has CLS symptoms on the leaves. Other potential sources of inoculum include asexual spores carried by wind^35, 39^ or insects^38, 39^ from neighbouring table beet or Swiss chard productions. Seedborne inoculum^32^ has also been suggested to contribute to CLS epidemics in some European countries^40, 41^ but its contribution to CLS epidemics in New York has not been investigated.

If the primary inoculum is introduced from a source outside a field of interest, such as infested seeds or windborne ascospores, we would expect to see a panmictic population with low population structuring due to frequent migration among fields. In fungal populations with long-distance dispersal of ascospores, a pattern of isolation by distance (IBD) is usually detected, resulting from the gradual spread of the disease from an original source of inoculum, hence, resulting in an increase in genetic differentiation among populations as distance increases^42, 43^. Alternatively, if a local source of inoculum, *e.g*., locally-developed sexual or asexual structures or infected weeds, are the major source of primary inoculum, genetic structuring of *C. beticola* populations would be present.

Potential survival of *C. beticola* on weeds or other crops could have profound impacts on CLS epidemiology as it would promote genetic diversity by reducing the impact of genetic drift through increasing the effective pathogen population size. Moreover, this would reduce the amount of disruptive selection pressure that the pathogen would have undergone by switching from a pathogenic to saprophytic mode.

If *C. beticola* inoculum surviving on local weeds or other susceptible crops (*e.g.* Swiss chard) is a major contributor to CLS epidemics on table beet, we would expect sympatric populations isolated from these alternate hosts to be genetically undifferentiated from *C. beticola* populations on table beet. To test this hypothesis, we examined *Cercospora* spp. populations from symptomatic lambsquarters growing sympatrically with table beet. Also, we genotyped *C. beticola* populations from Swiss chard and table beet plants growing in sympatry in mixed-cropping farms and gardens in New York and Hawaii, to investigate whether diseased Swiss chard may be a source of primary inoculum initiating CLS epidemics on table beet.

The objectives of this study were to i) investigate the potential for sexual reproduction in *C. beticola* populations in New York; ii) test the hypothesis that inoculum from lambsquarters and Swiss chard may contribute to CLS epidemics on table beet; and iii) determine the distribution of genetic diversity in *C. beticola* populations within and among mixed-cropping and monoculture table beet production to understand the relative contributions of local or external sources of inoculum to CLS epidemics. Herein, the term ‘population’ is used to refer to a group of isolates defined under certain geographical or host criteria (e.g., collected from one field/state or from the same host/variety).

## Results

### *Cercospora beticola* isolation, identification and genotyping

**Figure 1:**
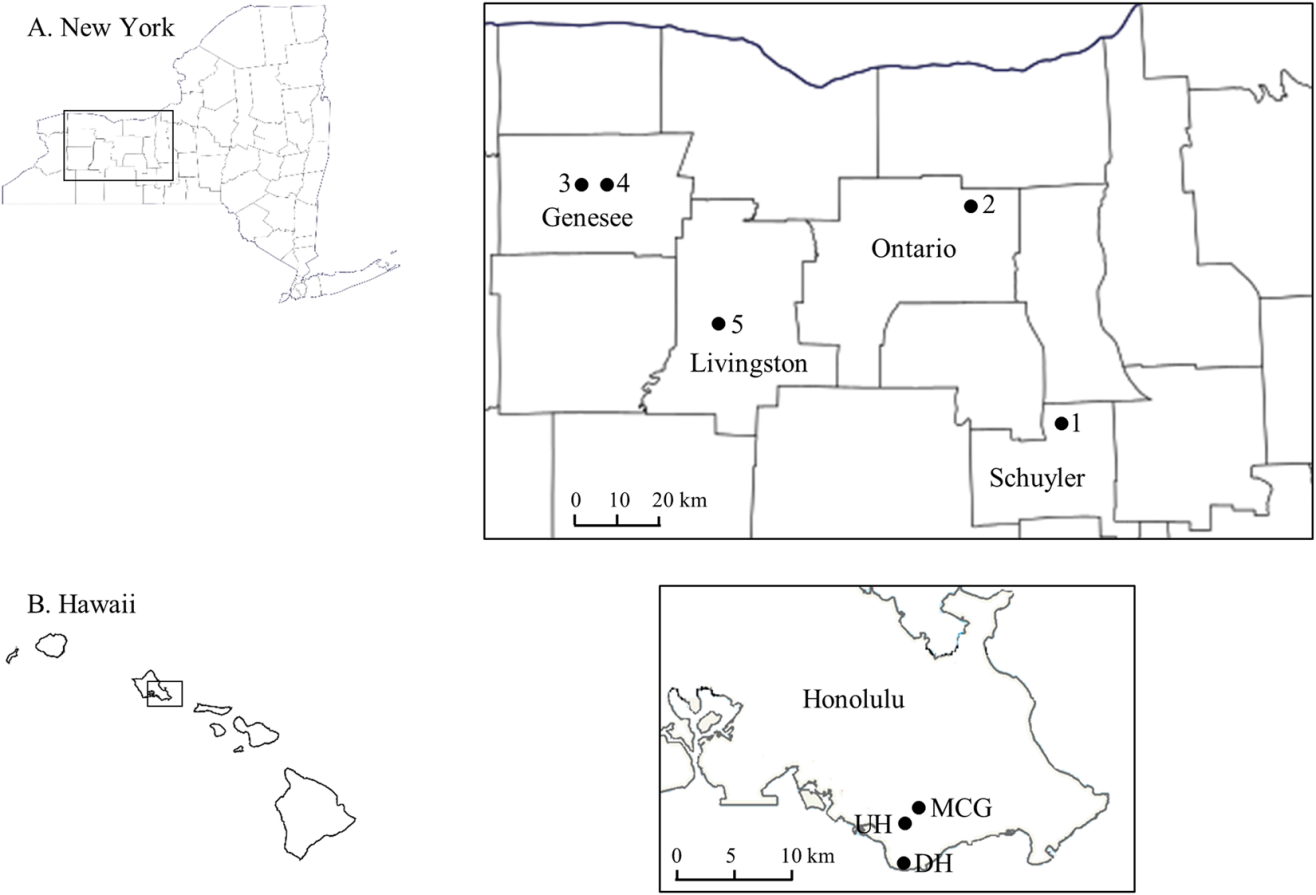
Sampling locations for *Cercospora beticola* populations collected in 2015 from (**A**) New York and (**B**) Hawaii. Numbers 1, 2, 3, 4, and 5 in New York represent Farm 1, Farm 2, Field 3, Field 4 and Field 5, respectively. In Hawaii, MCG, UH and DH represent community gardens in Honolulu (MCG = Manoa community garden, UH = University of Hawaii organic garden, and DH = Diamond Head community garden). The map presented here are modified from https://commons.wikimedia.org/wiki/File:Map_of_New_York_County_Outlines.svg and https://upload.wikimedia.org/wikipedia/commons/e/e5/Interstate_H1_map.png. Figures were produced in Microsoft Paint and Microsoft PowerPoint 2013.

In New York, 649 *C. beticola* isolates were obtained from 426 table beet and 14 Swiss chard plants collected from two mixed-cropping farms (Farms 1 and 2) and three monoculture table beet fields (Fields 3, 4 and 5), in 2015 (Fig. 1). Of these, 422 isolates were randomly selected for microsatellite genotyping and mating-type determination (Table 1). In Hawaii, 178 isolates were obtained from 34 and 65 table beet and Swiss chard plants, respectively, all of which were included for genotyping and mating-type determination (Table 1). Twenty-eight *Cercospora* isolates were obtained from symptomatic lambsquarters plants inter-mixed with table beet in Field 5. Morphological and cultural features of these isolates were distinct from *C. beticola* as conidiospores were shorter, subcylindrical in shape, straight to slightly curved; and also colonies on potato dextrose agar (PDA) were slow growing (~10 mm diameter after 14 days). Further multi locus sequence typing (ITS, actin, histone H3, translation elongation factor 1-α, and calmodulin) showed that these isolates belonged to *C. chenopodii* (*data not shown*), hence, were excluded from subsequent analyses. *Cercospora beticola*-specific primers (CercoCal-beta and CercoCal-R) resulted in a ~170 bp product in all *C. beticola* isolates from table beet and Swiss chard as well as *C. chenopodii* isolates from lambsquarters. This demonstrated that the primers CercoCal-beta and CercoCal-R were not specific to *C. beticola*. On the other hand, none of the microsatellite loci amplified a band in *C. chenopodii* (*data not shown*). Re-genotyping 25% of isolates detected no genotyping error except for one primer pair (CbSSR3), which had an error rate of 0.01.

**Table 1:** Sampling locations and hosts of *Cercospora beticola* isolates collected in 2015, and the total number of isolates genotyped (N_g_).

Field	State	Production type	Host	Variety	N_g_
Farm 1	New York	Mixed-cropping farm	Table beet	Detroit	48
Farm 2	New York	Mixed-cropping farm	Table beet	Detroit	75
			Table beet	Touchstone Gold	61
			Swiss chard	Oriole and Ruby Red	34
Field 3	New York	Monoculture	Table beet	Ruby Queen	70
Field 4	New York	Monoculture	Table beet	Ruby Queen	61
Field 5	New York	Monoculture	Table beet	Red Ace	73
DH	Hawaii	Mixed-cropping garden	Table beet	Unknown	41
			Swiss chard	Unknown	43
UH	Hawaii	Mixed-cropping garden	Swiss chard	Unknown	32
MCG	Hawaii	Mixed-cropping garden	Table beet	Unknown	20
			Swiss chard	Unknown	42
Total					600

### Within-plant and within-lesion genotypic diversity

Two to four distinct MLGs were obtained from six of the 12 intensively sampled plants. Isolations from multiple lesions on single leaves detected two to four distinct MLGs in 12 of 21 leaves (57%), which, in 50% of the leaves, also belonged to different mating-types. Up to three distinct MLGs, showing variation at two to 10 loci, were associated with single CLS lesions on table beet and Swiss chard plants. In one lesion, distinct MLGs belonged to different mating-types.

### Lack of host association in table beet and Swiss chard populations

**Figure 2:**
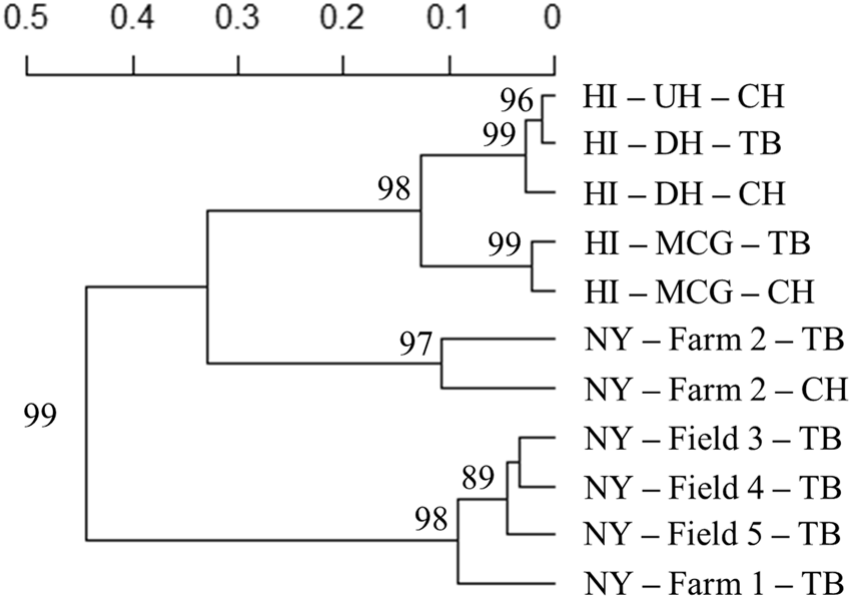
UPGMA dendrogram constructed based on Nei’s distance among *Cercospora beticola* populations from New York and Hawaii (MCG = Manoa community garden, UH = University of Hawaii organic garden, and DH = Diamond Head community garden), 2015, represented by state–field–host (TB = table beet and CH = Swiss chard).

At Farm 2, sympatric *C. beticola* populations collected from two varieties of table beet (‘Detroit’ and ‘Touchstone Gold’) were not genetically differentiated (Φ_ST_ = 0.002, *P* = 0.276; D = 0.007, 95% CI: 0.000–0.016; ${{\rm{G}}}_{\mathrm{ST}}^{^{\prime} }$ = −0.0134, CI: −0.020–−0.009). Moreover, no significant difference was detected in the genotypic diversity (*P* > 0.29) or genotypic composition (*P* = 0.19). From 20 recurrent MLGs at Farm 2, 13 were shared between these two table beet varieties. Likewise, sympatric populations from table beet and Swiss chard did not show significant population differentiation (Table 2). This was also demonstrated from the UPGMA dendrogram computed based on Nei’s distance (Fig. 2), in which sympatric populations from table beet and Swiss chard mostly grouped closer together, but more distant from populations from other fields. Therefore, for subsequent analyses, isolates from different hosts in one farm/garden were pooled into one population representing the corresponding farm/garden.

**Table 2:** Indices of differentiation for *C. beticola* populations collected from table beet and swiss chard growing in sympatry in New York (Farm 2) and Hawaii (DH and MCG). *P* values and confidence intervals were estimated after 999 randomisations.

Index of differentiation	Φ_PT_^49^ (*P* value)	Jost’s D^83^ (95% CI)	Hedrick’s ${{\rm{G}}}_{{\rm{ST}}}^{^{\prime\prime} }$ ^84^ (95% CI)
Field			
Farm 2 (*n* = 131)	0.000 (0.28)	0.007 (0.000 – 0.016)	−0.013 (−0.019 – 0.009)
DH (*n* = 69)	−0.095 (0.58)	0.051 (0.030 – 0.073)	−0.042 (−0.058 – −0.030)
MCG (*n* = 50)	−0.202 (0.95)	0.050 (0.025 – 0.080)	−0.056 (−0.081 – −0.037)

### Indices of genetic diversity

The number of alleles in the entire *C. beticola* population ranged from 2 (CbSSR2) to 24 (CbSSR3) with an average of 6.75 alleles per locus. Nei’s unbiased gene diversity (H_e_) per locus ranged from 0.034 (CbSSR1) to 0.87 (CbSSR3). In New York, locus CbSSR24 was monomorphic in Farm 1, while CbSSR1 was monomorphic in the other four locations. All loci were polymorphic for *C. beticola* populations from Hawaii (Table 3). Overall, allelic richness; private allelic richness; and allelic and genotypic diversity after rarefaction was higher in the *C. beticola* population from New York compared to Hawaii (Table 3).

**Table 3:** Indices of genetic diversity for *Cercospora beticola* populations collected from New York and Hawaii, 2015.

Population	N^a^	N_a_^b^	R_a_^c^	P_a_^d^	P_rare_^e^	%P^f^	NI loci^g^	H_e_^h^	MLG^i^	eMLG^j^	λ^k^	E_5_^l^	CF^m^	PrCp^n^		
														Obs.	Exp.	*P* value
Farm 1	34	2.8	2.6	5	0.47	91.7	CbSSR24	0.29	10	8.4	0.57	0.42	0.67	1.00	0.48	<0.01
Farm 2	131	4.1	3.1	4	0.22	91.7	CbSSR1	0.40	65	17.4	0.95	0.70	0.50	0.26	0.00	<0.01
Field 3	69	4.5	3.7	3	0.34	91.7	CbSSR1	0.40	40	20.6	0.98	0.86	0.51	0.53	0.21	<0.01
Field 4	59	3.7	3.3	1	0.12	91.7	CbSSR1	0.46	29	17.4	0.96	0.77	0.42	0.35	0.23	<0.01
Field 5	66	3.2	2.7	1	0.14	91.7	CbSSR1	0.37	22	13.3	0.90	0.70	0.71	0.80	0.32	<0.01
**New York**	**359**	**6.3**	**5.8**	**39**	**2.73**	**100**	**—**	**0.57**	**130**	**77.3**	**0.98**	**0.65**	**0.64**	**0.06**	**0.00**	<0.01
DH	69	2.8	2.4	2	0.06	100	—	0.31	8	5.0	0.54	0.55	0.88	0.91	0.25	<0.01
UH	26	2.5	2.5	0	0.00	75	—	0.31	6	6.0	0.70	0.71	0.77	1.00	0.60	<0.01
MCG	50	3.1	2.8	1	0.11	100	—	0.45	10	7.0	0.75	0.67	0.80	0.80	0.24	<0.01
**Hawaii**	**145**	**3.5**	**3.5**	**4**	**0.45**	**100**	**—**	**0.41**	**16**	**16.0**	**0.78**	**0.63**	**0.90**	**0.64**	**0.00**	<0.01

^a^N = population size after clone-correction to leaf level; ^b^N_a_ = mean number of alleles per locus; ^c^R_a_ = allelic richness after rarefaction to the smallest sample size; ^d^P_a_ = number of private alleles; ^e^P_rare_ = number of private allele after rarefaction to the smallest sample size; ^f^%P = percent polymorphic loci defined as loci with a frequency of at least 0.01; ^g^NI loci = non-informative (monomorphic) loci; ^h^H_e_ = Nei’s index of gene diversity defined as the probability that two randomly chosen alleles are different; ^i^MLG = number of multi-locus genotypes: MLGs in the total population is lower than the sum of MLGs of all populations because multiple MLGs occurred in more than one population, ^j^eMLGs = expected number of MLGs after rarefaction; ^k^ **λ = **Simpson’s complement index defined as the probability that two genotypes randomly chosen from the population are different; ^l^E_5_ = population evenness which is an estimation of the uniformity in distribution of the MLGs; ^m^CF = clonal fraction; ^n^PrCP = proportion of phylogenetically compatible pairs of loci, where Obs. denotes the observed value and Exp. denotes the PrCP value expected from a randomly recombined data set.

### Multi-locus genotype analyses

**Figure 3:**
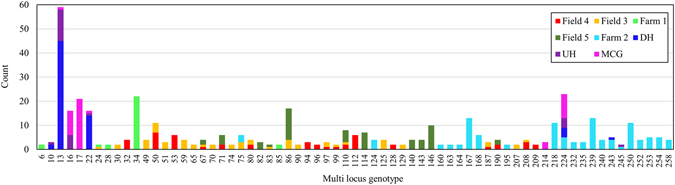
Recurrent multi-locus genotypes and their frequency in *Cercospora beticola* populations from New York (Farm 1, Farm 2, Field 3, Field 4 and Field 5) and Hawaii (MCG = Manoa community garden, UH = University of Hawaii organic garden, and DH = Diamond Head community garden), 2015.

**Figure 4:**
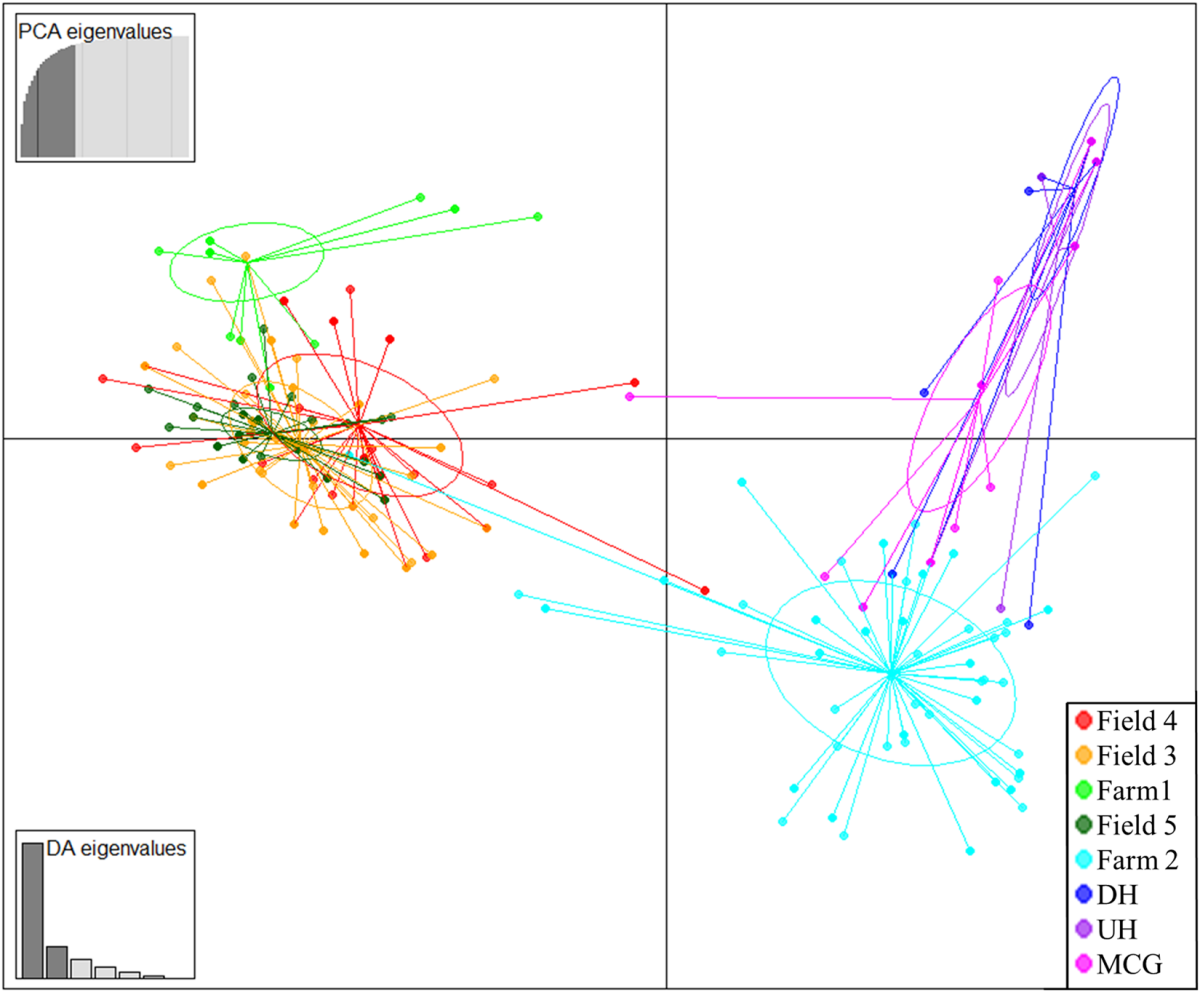
Discriminant Analysis of Principal Components for *Cercospora beticola* populations from New York (Farm 1, Farm 2, Field 3, Field 4 and Field 5) and Hawaii (MCG = Manoa community garden, UH = University of Hawaii organic garden, and DH = Diamond Head community garden), 2015.

In the 359 *C. beticola* isolates from New York, 130 unique MLGs were detected, resulting in 64% clonality of the population. Despite the relative high level of clonality in New York table beet fields, genotypic diversity was also high (λ ≥ 0.90) at all locations except for Farm 1, which had a moderate genotypic diversity (λ = 0.57). Each field contained a different dominant MLG (Fig. 3). For example, at Farm 1, MLG34 occurred more than 20 times in the population, which also had low genotypic evenness (Table 3). Of the 130 MLGs detected in New York, 60 MLGs were recurrent (occurred at least twice), and 14 were shared among fields (Fig. 4). Field 3 had the highest number of shared MLGs (12) while only one MLG from fields Farm 1 and 2 were present in another population (shared with Field 3).

In Hawaii, only 16 unique MLGs were detected among the 145 isolates, hence the 90% clonal fraction of *C. beticola* population (Table 3), which suggested the presence of a few dominant MLGs in the Hawaiian population (Fig. 3). Five MLGs were shared among locations in Hawaii, three of which occurred in all gardens. Only three MLGs were shared between New York and Hawaii, all of which occurred at Farm 2 (Fig. 3).

Farm 2 contained 20 recurrent MLGs, five of which occurred on both table beet and Swiss chard. In Hawaii, of the five recurrent MLGs in DH and MCG, three MLGs existed on both table beet and Swiss chard (Fig. S1). MLG245 occurred once in New York on table beet and once in Hawaii on Swiss chard.

For the 60 and 16 recurrent MLGs in New York and Hawaii, respectively, the probability of the MLGs having originated from independent sexual events was greater than 0.05 for only two and three MLGs (MLG80 and MLG207 for New York; and MLG10, MLG13 and MLG16 for Hawaii), confirming the clonal origin of the majority of the overrepresented MLGs.

### Population structure and differentiation

AMOVA analysis of the entire population found 25% of the total observed genetic diversity was partitioned among the fields within states (*P* = 0.001), 65% among individuals within fields (*P* = 0.001), and 10% between the two states (*P* = 0.15). Similar results were obtained through AMOVA analysis using R_ST_, where 20 and 80% of the total diversity existed among and within fields, respectively (*P* < 0.001), and none between New York and Hawaii (*P* = 1.00).

The pairwise index of differentiation Φ_PT_ showed significant differentiation among the five fields within New York (*P* < 0.013) except for Fields 3 and 4, which were not significantly differentiated (*P* = 0.715) (Table 4). The three monoculture fields (Fields 3, 4 and 5) showed low population differentiation (Φ_PT_ < 0.042, *P* < 0.05) while the two mixed-cropping farms (Farm 1 and 2) showed greater differentiation from each other (Φ_PT = _0.240, *P* < 0.05) and from the monoculture fields (Φ_PT_ > 0.137, *P* < 0.05) (Table 4).

**Table 4:** Pairwise index of population differentiation (Φ_PT_) between *Cercospora beticola* populations collected from New York (Farm 1, Farm 2, Field 3, Field 4 and Field 5) and Hawaii (DH, EH, and MCG), 2015. Φ_PT_ for the clone-corrected data set (indicated in parentheses) was not calculated for the Hawaiian populations due to the small population sizes. Significant differentiation (*P < *0.05) is indicated in bold.

	Field 3	Field 4	Field 5	Farm 1	Farm 2	DH	UH
Field 4	0.023 (0.012)						
Field 5	**0.042 (0.025)**	**0.035 (0.033)**					
Farm 1	**0.330 (0.230)**	**0.137 (0.173)**	**0.318 (0.165)**				
Farm 2	**0.256 (0.431)**	**0.188 (0.398)**	**0.191 (0.402)**	**0.240 (0.326)**			
DH	**0.095**	**0.107**	**0.213**	**0.358**	**0.424**		
UH	**0.198**	**0.194**	**0.354**	**0.376**	**0.595**	0.024	
MCG	**0.202**	**0.193**	**0.336**	**0.313**	**0.521**	**0.103**	**0.099**

**Figure 5:**
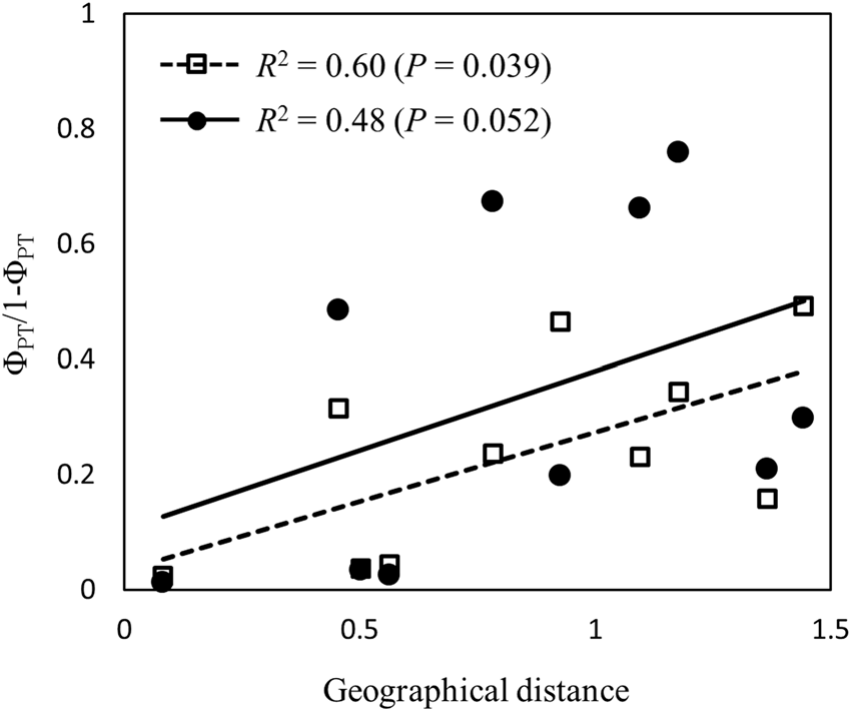
Standardized pairwise genetic distance (Φ_PT_/(1 − Φ_PT_)) plotted against geographic distance among five *Cercospora beticola* populations in New York, 2015. The open squares and dashed line are based on the complete data set, and the filled circles and solid line are based on the clone-corrected data set.

Discriminant Analysis of Principal Components (DAPC) using fields as pre-defined populations also demonstrated that populations from the three monoculture table beet fields overlapped while those from mixed-cropping farms clustered separately (Fig. 4). The Mantel test detected significant association between the genetic and geographical distance of the five *C. beticola* populations in New York for the entire dataset (*R* ^2^ = 0.60, *P* = 0.039) but not for the clone-corrected data set (*R* ^2^ = 0.48, *P* = 0.052) (Fig. 5).

**Figure 6:**
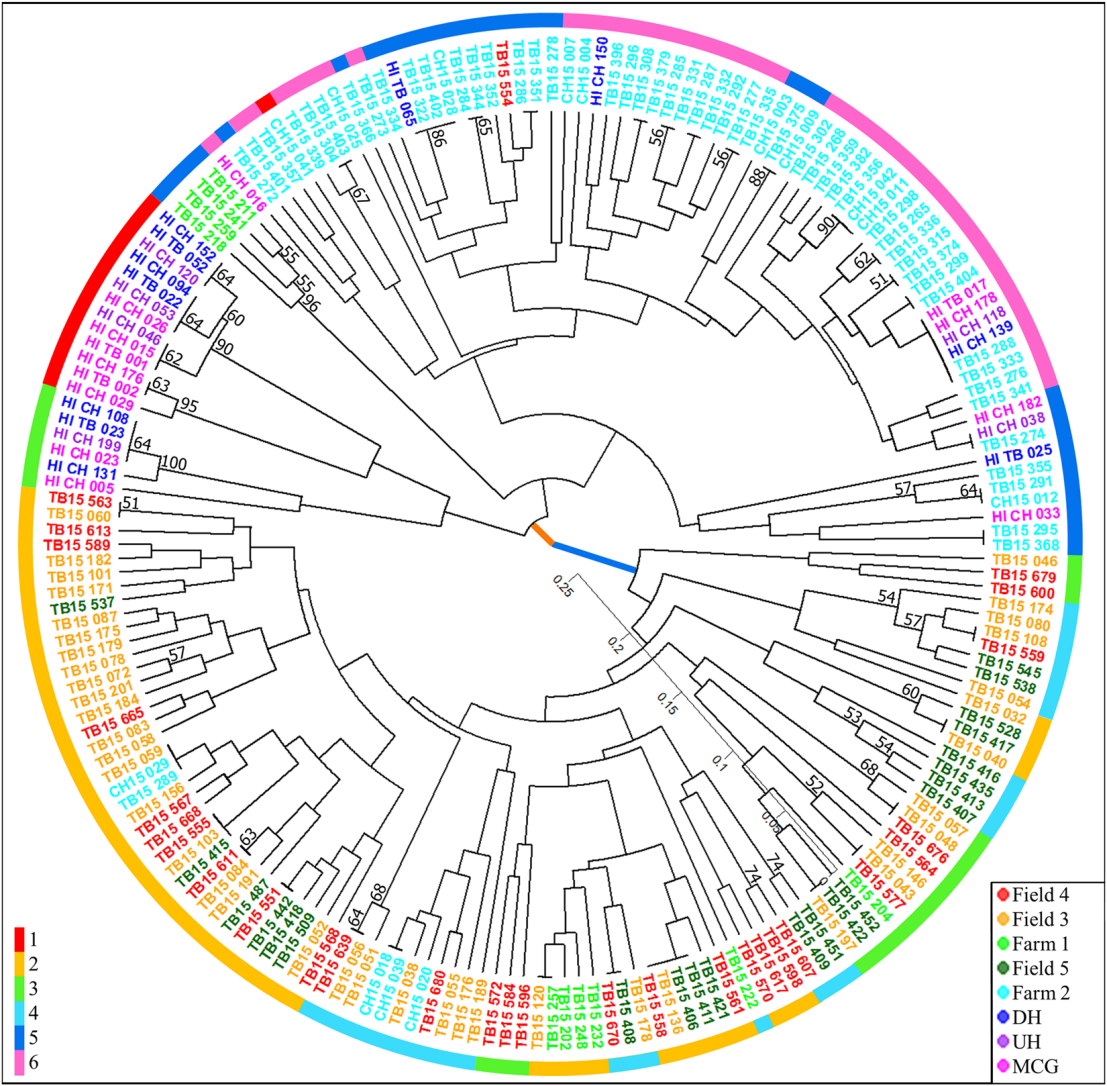
UPGMA dendrogram of *Cercospora beticola* isolates collected in 2015 from New York (Farm 1, Farm 2, Field 3, Field 4 and Field 5) and Hawaii (MCG = Manoa community garden, UH = University of Hawaii organic garden, and DH = Diamond Head community garden) based on Bruvo’s distance. Bootstrap support values greater than 50 are shown above the branches. The thick orange and blue branches denote the two clusters detected through Bayesian clustering method implemented in STRUCTURE. The colour of the isolates correspond to the field/community garden they belong as indicated in the right-hand-side legend. The colour of the outer ring corresponds to the six clusters detected by DAPC analysis as shown in the left-hand-side legend and Fig. S3.

Population structure analysis with no *a priori* population assignment through Bayesian clustering analysis of both the clone-corrected and non-clone-corrected datasets resulted in detection of two distinct clusters in the population (Fig. S2). Likewise, a dendrogram produced based on Bruvo’s distance (Fig. 6) grouped the *C. beticola* isolates into two major clades. Two hundred and twenty-nine isolates were assigned to cluster 1 and 275 isolates were assigned to the remaining cluster. These two clusters did not correspond to sampling location or host (Fig. 6). DAPC analysis for the non-clone-corrected data set was inconclusive as the Bayesian Information Criterion (BIC) consistently decreased with the number of clusters until K reached the total number of MLGs. For the clone-corrected data set, however, an increase in BIC was observed with increasing number of K (Fig. S3). K = 6 was selected as the optimal number of clusters since the BIC value decreased by a negligible amount after K = 6 (Fig. S3). Three of these groups (1, 5 and 6) belonged to cluster 1 detected in STRUCTURE, group 1 and 4 belonged to cluster 2, while individuals in group 3 occurred in both the first and second clusters detected in STRUCTURE.

### Mating-type determination

**Figure 7:**
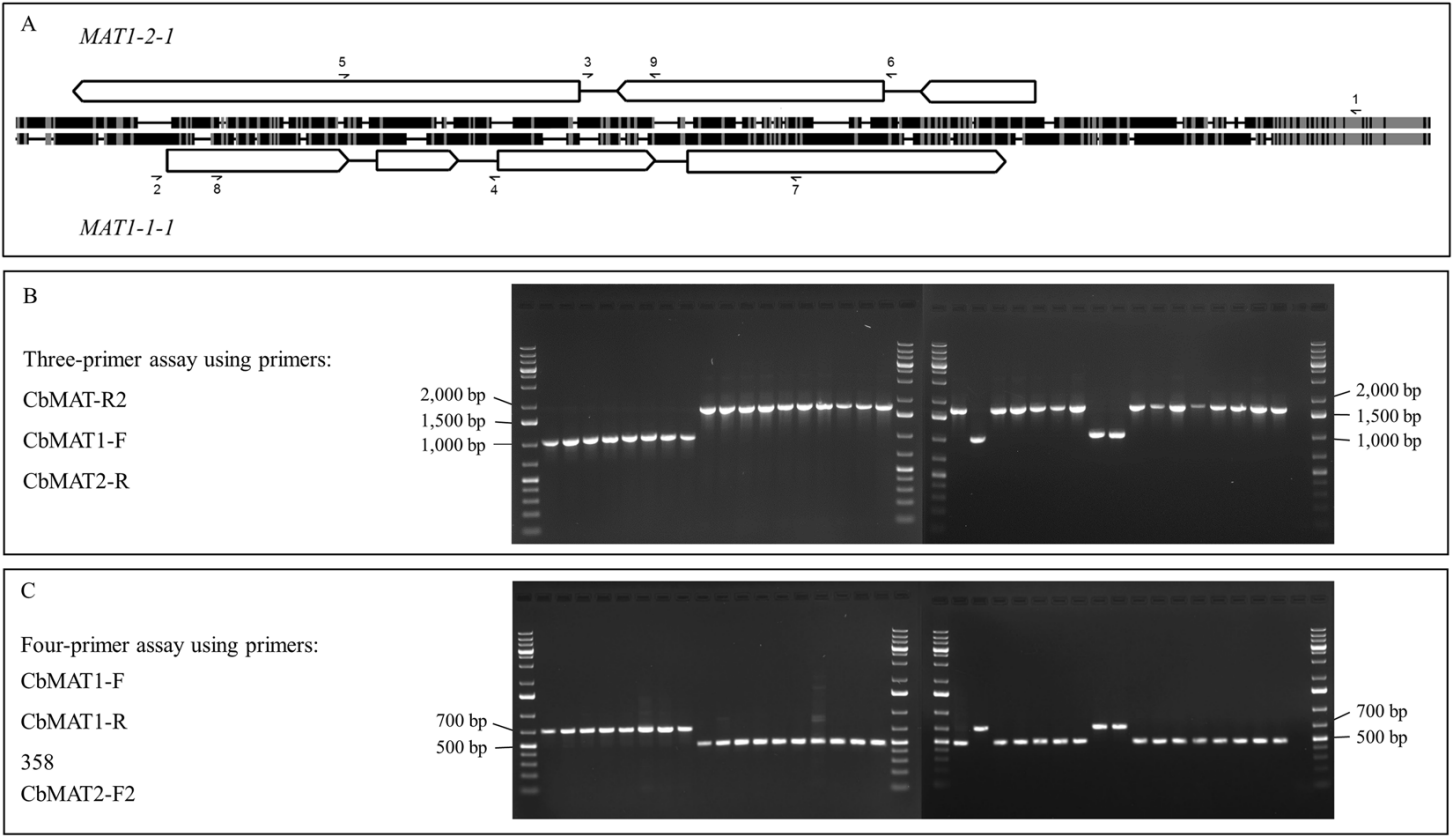
(**A**) Schematic alignment of the *MAT1* idiomorphs in *Cercospora beticola; MAT1-2-1* (top) and *MAT1-1-1* (bottom), showing the position and orientation of the open reading frames with arrowed boxes, and position of introns with black lines. The position of the primers used for mating-type determination are shown with arrows bearing the primer number according to Table 5. The thick black and grey lines in the alignment represent differences and similarities, respectively. (**B**) Three-primer mating-type assay, resulting in PCR amplicons of ~1,700 and ~1,050 bp in MAT1-1 and MAT1-2 isolates, respectively. (**C**) Four-primer mating type assay, resulting in PCR amplicons of ~500 and ~700 bp in MAT1-1 and MAT1-2 isolates, respectively. The first and last line in each gel include 6 µL of O’GeneRuler 1 kb Plus DNA Ladder (Thermo Scientific).

Screening all isolates by multiplex PCR using primers CercosporaMat1F/R and 356/358 for mating-type determination (Table 5) resulted in multiple, non-specific PCR products in ~30% of the isolates, which confused mating-type determination (*data not shown*). The novel three-primer and four-primer mating-type assays developed based on non-exonic regions of the mating-type genes (Fig. 7; Table 5) successfully amplified a single band of the expected size in these isolates.

**Table 5:** Multiplex PCR assays and primers used for mating-type amplification and determination in *Cercospora beticola* populations. References are provided for the primers that were not designed in this study.

Assay	Primer name	Primers sequence (5′ to 3′)	Expected amplicon size
1	1: CbMAT-R2	GCT GCA TTG ACT GAC TGT CG	~1,700 bp for MAT1-1 and ~1,050 bp for MAT1-2 isolates
	2: CbMAT1-F	AAC AAT CGG ATC CAC TAC CG	
	3: CbMAT2-R	CAA GCT CAT GAA GTC AGA GAT ACG	
2	2: CbMAT1-F	AAC AAT CGG ATC CAC TAC CG	~500 bp for MAT1-1 and ~700 bp for MAT1-2 isolates
	4: CbMAT1-R	GCA AGA AGA GCC CTG TCA AG	
	5: 358^28^	CTG TGG AGC AGT GGT CTC	
	6: CbMAT2-F2	ATC ATC ACT AAC ATC TTG AAG GGC TA	
3^28^	5: 358^28^	CTG TGG AGC AGT GGT CTC	~800 bp for MAT1-1 and ~400 bp for MAT1-2 isolates
	7: CercosporaMat1-R^26^	GAG GCC ATG GTG AGT GAG	
	8: CercosporaMat1-F^26^	CTT GCA GTG AGG ACA TGG	
	9: 356^28^	GAT CTA CCG TCT CGA CCT C

### Tests for random mating

#### Mating-type ratios

For the non-clone-corrected data set, mating-type frequencies deviated from a 1:1 ratio expected under random mating for all populations except for that derived from Farm 2 (Table 6). After clone-correction, MAT1-1 and MAT1-2 isolates were present in equal ratios in all fields except for 4 and 5 in New York (Table 6).

**Table 6:** Tests of linkage equilibrium of microsatellite loci and mating-type frequencies for *Cercospora beticola* populations collected from New York and Hawaii, in 2015.

Population	Non-clone-corrected			Clone-corrected			Non-clone-corrected		Clone-corrected	
	*MAT1-1*	*MAT1-2*	χ^2a^	*MAT1-1*	*MAT1-2*	χ^2a^	$\overline{{{\boldsymbol{r}}}_{{\boldsymbol{d}}}}$ ^b^	*I*_*a*_^c^	$\overline{{{\boldsymbol{r}}}_{{\boldsymbol{d}}}}$ ^b^	*I*_*a*_^c^
Farm 1	7	27	**11.77**	5	5	n.a.^d^	**0.65**	**6.43**	**0.51**	**5.04**
Farm 2	63	68	0.19	28	40^f^	2.11	**0.14**	**1.42**	**0.13**	**1.34**
Field 3	44	24^e^	**5.88**	26	14	3.60	**0.04**	**0.38**	**0.03**	**0.26**
Field 4	47	12	**20.76**	22	7	**7.76**	**0.08**	**0.81**	**0.04**	**0.35**
Field 5	54	12	**26.73**	19	3	**11.64**	**0.10**	**0.95**	**0.05**	**0.48**
New York	215	143	**14.48**	97	68	**5.10**	**0.164**	**1.73**	**0.14**	**1.54**
DH	16	53	**19.84**	4	7	n.a.^d^	**0.50**	**5.24**	**0.18**	**1.87**
UH	2	24	**14.29**	2	4	n.a.^d^	**0.60**	**6.26**	**0.31**	**3.26**
MCG	5	45	**32.00**	3	10	3.77	**0.45**	**4.72**	**0.16**	**1.75**
Hawaii	23	122	**67.59**	9	21	**4.80**	**0.374**	**4.09**	**0.21**	**2.31**
Total	238	265	1.449	106	89	1.482	**0.161**	**1.746**	**0.132**	**1.437**

^a^χ^2^ value based on 1:1 ratio and one degree of freedom. Values related to mating-type frequencies with significant deviation from one (*P* < 0·05) are indicated in bold; ^b^ $\bar{r}$_d_ = standardized index of association scaled for the number of loci with significant deviations from zero (*P* < 0.05) indicated in bold; ^c^ *I*_*a*_ = index of association with significant deviations from zero (*P* < 0.05) indicated in bold; ^d^n.a. = not applicable or not calculated due to small sample sizes; ^e^isolate Tb15-056 (from Field 3) failed to amplify a band after three PCRs, hence the total number of isolates from this field was 68 (rather than 69); ^f^three pairs of isolates with identical microsatellite genotypes in Farm 2 belonged to alternate mating-types, hence the total isolates for the clone corrected data set is 68, which was larger than the number of MLGs in this farm (65).

#### Linkage disequilibrium of microsatellite loci

Significant linkage of microsatellite loci was detected in all populations in New York and Hawaii even after clone-correction of datasets, rejecting the null hypothesis of random mating (Table 6).

Analyses of mating-type ratios and linkage disequilibrium of microsatellite loci conducted for the two clusters detected through Bayesian clustering implemented in STRUCTURE also rejected the null hypothesis of random mating (Table 7).

**Table 7:** Tests of linkage equilibrium of microsatellite loci and mating-type frequencies for two *Cercospora beticola* clusters detected through Bayesian clustering using the software STRUCTURE. Significant values (*P* < 0.05) are indicated in bold.

Population	Non-clone-corrected					Clone-corrected				
	*MAT1-1*	*MAT1-2*	χ^2^	$\overline{{{\boldsymbol{r}}}_{{\boldsymbol{d}}}}$	*I*_*a*_	*MAT1-1*	*MAT1-2*	χ^2^	$\overline{{{\boldsymbol{r}}}_{{\boldsymbol{d}}}}$	*I*_*a*_
Cluster 1	150	79	**22.013**	**0.049**	**0.509**	69	32	**13.554**	**0.030**	**0.306**
Cluster 2	88	186	**35.051**	**0.231**	**2.485**	37	57	**4.255**	**0.140**	**1.493**

### Test for recombination

The proportion of compatible loci (PrCP) was less than one in four sampling locations in New York and in two locations in Hawaii (Table 3). The observed PrCP values in all these locations were significantly larger than expected under the null hypothesis of random mating. Analysis of the phylogenetically compatible pairs of loci for the two clusters detected through Bayesian clustering implemented in STRUCTURE indicated that only 36 and 40% of the pairs of loci were compatible in cluster 1 and 2, respectively. For both clusters, the observed value of PrCP was significantly larger than expected under random mating (*P* < 0.001).

## Discussion

To enhance our understanding of the epidemiology and sources of inoculum contributing to CLS epidemics on table beet, 12 microsatellite markers were used to characterise the genetic structure of *C. beticola* populations in contrasting table beet production systems in New York. Sympatric *Cercospora* spp. populations collected from table beet, Swiss chard and lambsquarters in New York and Hawaii were also studied to establish whether inoculum from alternative hosts contribute to CLS epidemics on table beet.

*Cercospora beticola* is known to infect a wide range of plants from various families^32, 38, 44–48^. Thus, infected weeds, especially from the family *Amaranthaceae*, are believed to serve as *C. beticola* inoculum reservoirs. One of these species is lambsquarters, which was found to be susceptible to *C. beticola* in glasshouse pathogenicity trials (*data not shown*). However, all *Cercospora* spp. collected from symptomatic lambsquarters plants growing in sympatry with table beet were identified as *C. chenopodii*. This is congruent with reports that studies in the Netherlands in 2003 also failed to isolate *C. beticola* from weeds^49^; and emphasises the need for more comprehensive surveys on a range of weeds from *Amaranthaceae* and other families to better understand the prevalence and incidence of *C. beticola* on hosts other than *Beta vulgaris*.

Sympatric *C. beticola* populations from different table beet varieties were not genetically differentiated based on microsatellite allele frequencies, nor did they differ in genotypic diversity or composition. Likewise, sympatric populations from Swiss chard and table beet in New York and Hawaii were not genetically differentiated. However, populations from Swiss chard and table beet from a mixed-cropping farm (Farm 2) in New York were significantly different in genotypic composition. Of the 20 recurrent MLGs in this farm, 13 were shared between the two table beet varieties while only five were shared between Swiss chard and table beet. This may be attributed to differences in virulence and/or aggressiveness of these MLGs on different hosts. For example, *Melampsora larici-populina* populations from cultivated and wild poplars showed little population differentiation based on neutral genetic markers while displaying different virulence profiles^42^. The lack of genetic differentiation of *C. beticola* populations from Swiss chard and table beet based on microsatellite allele frequencies is suggestive of lack of reproductive isolation between these populations. Thus, infected Swiss chard from nearby organic productions or home gardens may potentially serve as a source of inoculum for CLS epidemics in table beet fields.

In 50% of intensively sampled plants and leaves, multiple unique MLGs were detected on the same plant or leaf. Single lesions were also occasionally found to be associated with up to three unique MLGs. Co-infection of a single lesion with multiple MLGs has been reported in other pathosystems^50, 51^, including *C. beticola* on sugar beet in Europe^41^. Moretti *et al*. used Random Amplified Polymorphic DNA (RAPD) and Direct Amplification of Minisatellite region DNA (DAMD) to characterize 10 *C. beticola* isolates obtained from a single lesion on sugar beet, all of which showed distinct banding patterns^41^. As the arrangement of the mating-type genes in *C. beticola* indicates heterothallism^26, 27^, the co-occurrence of multiple MLGs in the same lesion may have profound impacts on population structure of this pathogen by enabling MLGs with alternate mating-types to sexually reproduce. Detection of two alternate mating-types in a single lesion in this study provides further evidence of the potential of *C. beticola* to undergo sexual reproduction.

High genotypic diversity, detection of admixed genotypes by Bayesian clustering and DAPC analyses (Figs S2 and S3), and lack of congruence between the clusters produced in UPGMA and DAPC analyses (Fig. 6), are all suggestive of recombination in the *C. beticola* population. Although the potential of rare parasexual events, somatic recombination or mitotic crossing over^20, 52, 53^ cannot be disregarded, the presence of mating-types in equal ratios is some table beet fields suggests that some *C. beticola* populations may be sexually reproducing.

On the other hand, overrepresentation of MLGs (high clonality), unequal frequency of mating-types in some fields, and linkage disequilibrium of microsatellite loci in all populations, even after clone correction, rejected the null hypothesis of random mating. Since other phenomena such as population structure/admixture can also result in linkage disequilibrium of loci^20^, tests of random mating were conducted on the two major clusters detected using Bayesian analyses (Table 7), which again rejected the null hypothesis of random mating. Severe bottlenecks and founder events, which occur frequently in agroecosystems, cause random changes in pathogen populations, which may result in linkage disequilibrium (LD) of loci^54^. Moreover, unequal frequency of mating-types and significant LD only reject the null hypothesis of random mating but do not prove the lack of sex as many fungal populations with the ability to sexually reproduce have been reported to have a clonal structure^55, 56^. Since most plant pathogenic fungi undergo clonal reproduction at some stage in their life cycles, deviations from random mating are expected and do not indicate lack of recombination^54^.

To test the hypothesis of strict clonality, rather than random mating, we tested the presence of phylogenetic incompatibility between pairs of loci. Under the assumption that recurrent, parallel and reverse mutations are highly improbable, phylogenetic incompatibility of loci may only arise by recombination^54^. Hence, the fact that 47, 65, 20 and 74% of pairs of loci were incompatible in Fields 3, 4, 5, and Farm 2 may be interpreted as presence of recombination in these populations. Although it is plausible that high levels of homoplasy, which is a known drawback of microsatellite loci^57^, contributed to the low rate of phylogenetic compatibility, the possibility of recombination cannot be disregarded.

Multiple studies have suggested that introduction of inoculum to initiate CLS epidemics occurs through rain splash dispersal of conidiospores from infected plant residues from previous years^32, 33, 58^. Survival of *C. beticola* inoculum on plant debris within soil has been reported in many studies to be at least 10 months^35^ to a maximum of three years^36^, depending on the climatic conditions. Planting sugar beet seed in infested soil was reported to result in infection of cotyledons and young seedlings^33^. Infection through the roots of mature plants has also been reported in sugar beet^49, 59^ but could not be reproduced in subsequent studies^35^. Wilting of beet leaves under hot conditions in the field occurs frequently, bringing the leaves in contact with the soil^33^. The moisture trapped under the lower surface of the leaves may contribute to leaf infection from *C. beticola* inoculum in the surface of the soil^33^. However, the monoculture table beet fields in New York follow rotation guidelines with three to five^37^ years of non-host crops between table beet. This infers that after five years, infected plant debris must be present with sporulation close to the soil surface. Further studies are required to quantify the long-term survival of *C. beticola* on plant residues, or in soil^33^ in New York.

Low genetic differentiation among the monoculture table beet fields (Φ_PT_ ≤ 0.042), and lack of differentiation between Fields 3 and 4 was surprising. Fields 3 and 4 are more than six kilometres apart, and separated from Field 5 by at least 50 km. Considering the long rotation periods between table beet crops in processing, monoculture table beet fields, significant founder events leading to high genetic drift and stochastic differentiation of the *C. beticola* population may be expected^60^, unless external sources of inoculum dominate and homogenise these populations. In addition to the lack of genetic differentiation, Fields 3 and 4 also had eight MLGs in common, and each shared four MLGs with Field 5. Although this could be caused by the low power of the microsatellite loci to resolve these clones into distinct MLGs, distribution of these clones over processing fields can be indicative of genotype flow. CLS epidemics in fields where sugar beet had not been planted for 20 years in the Netherlands^61^ and table beet fields that had never been planted to table beet (George Abawi, *personal communication*), also suggest a means of long-distance dispersal for this pathogen.

A pattern of IBD is usually interpreted as the natural process of dispersal of windborne inoculum, resulting from the gradual spread of the disease from an original source of inoculum^42^. However, *C. beticola* conidiospores are believed to disseminate through rain splash^32, 39, 58^, and travel short distances (100 to 400 m) by wind^32, 34, 62^. Moreover, long-distance dispersal of ascospores, if present, cannot explain the dissemination of clones as *C. beticola* is heterothallic and potential sexual reproduction would not likely result in the production of clones. Plausible sources of long distance dispersal of the MLGs among the monoculture table beet fields are insects^32^, human movement of agricultural machinery or seed, the role of which in *C. beticola* dispersal requires further investigation. In this study, the seed for the three monoculture fields all originated from Mount Vernon, WA, USA.

Structuring of *C. beticola* populations from mixed-cropping farms in New York may be attributed to the dominant role of local sources of inoculum in initiating CLS epidemics in these fields. Populations from the two mixed-cropping farms in New York were highly differentiated from each other and also from the monoculture fields. Each of the two mixed-cropping farms contained a different dominant genotype (Fig. 3), and did not share genotypes with each other, indicative of limited dispersal between these fields. The seed for each of the mixed-cropping farms was obtained from different sources. Thus, if infested seed is a significant source of inoculum to CLS epidemics, the disparate population structure in the two mixed-cropping farms could have also been the result of seedborne inoculum, further emphasising the need for additional studies on the role of seed in CLS epidemics.

Future studies should quantify temporal changes in genotypic diversity and composition in mixed-cropping farms to enhance the understanding of the relative potential sources of inoculum to CLS epidemics. Moreover, population genomics studies and use of higher resolution molecular markers such as single nucleotide polymorphisms may have utility to improve our detection of recombination events or signs of sex in *C. beticola* populations in New York.

## Methods

### Sampling and isolation

Sampling of *Cercospora beticola* populations in New York was conducted in 2015 from two mixed-cropping farms (Farm 1 and Farm 2) and three monoculture table beet fields (Fields 3 and 4 = variety Ruby Queen; Field 5 = variety Red Ace) (Fig. 1). The mixed-cropping farms consisted of small-scale vegetable production producing a few rows of table beet, Swiss chard, and other fresh vegetables on an annual basis. Monoculture production consisted of large-scale (>0.2 km^2^) table beet fields with at least 3 years of a non-host crop prior to table beet cultivation.

Hierarchical sampling was conducted involving the arbitrary selection of sampling points at defined intervals along transects/rows, and sampling of multiple symptomatic plants within each location. The distance between the sampling points and number of plants sampled per location varied between the processing and fresh market production systems due to differences in the size of the fields. In the three monoculture fields, five sampling locations were selected at 20-m intervals along each of the two transects, which were separated by 50 m. Ten plants where arbitrarily sampled within a 5-m radius of each sampling location (*n* = 100 in each field). In addition, 28 symptomatic lambsquarters plants were collected from Field 5 to obtain a sympatric population of *C. beticola*. The mixed-cropping farms, each included only a few rows of table beet, which were intensively sampled at 5–10-m intervals along the rows. Farm 1 included three rows of table beet variety Detroit, from which 36 symptomatic plants were sampled. Farm 2 included three rows of table beet variety Touchstone Gold and one row of table beet variety Detroit as well as two rows of Swiss chard varieties Oriole and Ruby Red planted intermixed with each other. The incidence of CLS on the Swiss chard was substantially lower than on table beet at this location. Therefore, symptomatic leaves from 90 table beet and 14 Swiss chard plants were collected from Farm 2.

Sampling in Hawaii was conducted at two community gardens in Honolulu, and at the University of Hawaii at Manoa, in 2015 (Fig. 1). The community gardens consisted of small-scale plots of an average size of 10 × 15 m, planted to a range of vegetables in an intermixed manner. When Swiss chard or table beet plants with CLS symptoms were observed in these plots, 1–3 leaves per plant were sampled.

For all plants sampled in New York, only one *C. beticola* isolate per plant was obtained, except for 12 arbitrarily selected plants, from which multiple isolates were obtained from leaves of single plants, lesions on single leaves, and also from single lesions as described by Bolton *et al*.^63^. For Hawaiian samples, up to 10 leaves were arbitrarily chosen from each garden for intensive isolation, where multiple isolates were obtained from a single leaf, or a single lesion.

### *Cercospora beticola* identification and genotyping

Genomic DNA was extracted from all fungal isolates as described previously^37^, and the species-specific PCR primers CercoCal-beta and CercoCal-R^64^ were used to confirm the identity of isolates as *C. beticola*. Genotyping was done for 600 isolates (178 isolates from Hawaii and 422 from New York) using 12 microsatellite markers (CbSSR1, CbSSR2, CbSSR3, CbSSR6^65^, CbSSR20, CbSSR21, CbSSR22, CbSSR23, CbSSR24, CbSSR25, CbSSR26, and CbSSR27^66^) as described previously^66^. Alleles were scored using the Geneious Microsatellite Plugin v. 1.4^67^. To reduce genotyping error, genotyping was replicated for 25% of the samples, including all isolates showing null or rare alleles, and error rates^68^ (e_l_) were quantified for each locus. Genotyping was conducted a third time for samples that showed allelic variation in locus CbSSR3 between the two replications.

### Microsatellite dataset refinement

Intensive isolation of multiple *C. beticola* strains from single leaves or lesions may result in re-isolating the same individual multiple times, which may skew the result of population genetic analyses. Therefore, the multi-locus genotypes (MLGs) for all isolates were determined in the R package *poppr* v.2.2.0^69, 70^ to assess the occurrence of MLGs in single lesions and leaves. For subsequent genetic diversity and structure analyses, the data set was clone-corrected to the leaf level, *i.e*., identical MLGs isolated from a single lesion/leaf were identified and only one representative isolate from each leaf was retained for subsequent analyses. This resulted in 359 and 145 isolates from New York and Hawaii, respectively. In order to reduce the effect of genotyping error and missing data on genetic diversity, the data set was filtered in *poppr* using the Bruvo’s^71^ distance “farthest neighbour” algorithm, and filtering threshold estimated by the function cutoff_predict (0.02083333). All subsequent analyses were conducted on the filtered dataset.

### Measures of genetic diversity

Nei’s^72^ measure of allelic diversity and number of private alleles were estimated in GenAlEx^73, 74^. Allelic richness (R_a_) and private allelic richness (P_a_) were estimated with rarefaction in ADZE v. 1.0^75^. The number of multi-locus genotypes (MLGs), expected number of MLGs after rarefaction (eMLG), and evenness (E_5_) were estimated in *poppr*. Simpson’s^76^ complement index of genotypic diversity (λ; the probability that two randomly selected genotypes are different) was calculated in *poppr*, and was corrected for sample size by multiplying by N/(N-1). Statistical significance of the differences in genotypic diversities among populations was tested in GenoDive^77^ using 999 bootstraps. In order to investigate the sufficiency of the microsatellite loci to capture the genotypic diversity of the *C. beticola* populations, mean genotypic diversity was plotted against the number of loci using MultiLocus v.1.3^78^. Recurrent genotypes (MLGs that occurred more than once) and their frequencies across populations were obtained using *poppr*. The probability that recurring MLGs could have arisen independently through sexual reproduction was investigated by estimating *P*_sex_ in GenClone 2.0^79^, and statistical significance was computed by 999 randomisations.

### Population differentiation and spatial genetic structure

All analyses of population differentiation were conducted on *a priori* populations (defined as individuals from the same host/field/state) using both clone-corrected and non-clone-corrected datasets. Pairwise Φ_PT_^80^, an analog of F_ST_ assuming a stepwise mutation model, was calculated with 999 randomisations in GenAlEx^73, 74^. Jost’s^81^ measure of population differentiation (D) and Hedrick’s^82^ ${{\rm{G}}}_{{\rm{ST}}}^{\text{'}}$ were estimated using the package *mmod* ^83^ in R, with 95% confidence intervals estimated after 1,000 bootstrap simulations. IBD for the five fields within New York was investigated through regression analysis of standardised genetic distance (Φ_PT_/(1 − Φ_PT_)) plotted against the logarithm of geographical distance. The Mantel^84^ test was conducted using *ade4* implemented in *mmod* for both clone-corrected and non-clone-corrected data sets. Discriminant Analysis of Principal Components (DAPC)^85^ among pre-defined populations from fields were conducted in the R package *adegenet* v.2.0.1^86^. The optimal number of PCs to retain was estimated through cross-validation using the function xvalDapc. A dendrogram of genetic distance among populations was produced through the unweighted pair group method with arithmetic mean (UPGMA) method in *poppr* based on Nei’s^87^ distance. Analysis of molecular variance (AMOVA)^88^ was conducted using *ade4* implemented in *poppr* (999 permutations), and also by computing a distance matrix (R_ST_) in Arlequin^80^ (1,000 randomisations). For AMOVA analysis in *poppr*, the option of filtering with a threshold of 0.05 was used to correct for genotypes that had equivalent distance due to missing data.

### Population structure

The existence of an underlying structure without *a priori* assumption of populations was tested using three methods for both clone-corrected and non-clone-corrected datasets: the model-based Bayesian clustering method implemented in STRUCTURE v.2.3.4^89^, DAPC conducted in the R package *adegenet*, which does not require assumptions of linkage equilibrium of loci, and a distance-based dendrogram through UPGMA using Bruvo’s^71^ distance in *Poppr*. Using STRUCTURE, assignment of MLGs to clusters was inferred for one to 15 clusters. Each model was simulated 10 times with a burn-in period of 100,000 Monte Carlo Markov Chains, and a run length of 1,000,000 iterations. The optimal K was chosen by computing ΔK^90^ using STRUCTURE HARVESTER v.0.6.94^91^. The 15 replicated runs for the optimal K were combined and a single graphical output was generated using CLUMPAK^92^. DAPC analysis was conducted using *adegenet*, with the optimal number of clusters determined using the function find.clusters. DAPC was then used to assign individuals into clusters, retaining the number of principal components encompassing 89% of the cumulative variance. A distance-based dendrogram using Bruvo’s distance was plotted using *poppr* with 1,000 bootstrap replicates, and branch support values greater than 50% were retained.

### Mating-type determination

For mating-type determination, all isolates were initially screened in a multiplex PCR using CercosporaMat1F/R^26^ and 356/358^28^ primers. Isolates carrying the *MAT1-1* and *MAT1-2* alleles were expected to produce amplicons of 805 and 442 bp, respectively. However, in 30% of the isolates, multiple bands were amplified, which confused mating-type determination (*data not shown*). This could be due to the fact that *C. beticola* isolates, in addition to a complete *MAT1* idiomorph, contain exonic fragments of both *MAT1-1* and *MAT1-2* alleles in their genome^93^. Thus, to improve specificity of the assay, new PCR primers were designed to the non-exonic regions of the *MAT1* alleles (Fig. 7; Table 5), which were further used to determine the mating-type of the remaining isolates. Two assays were developed: 1) a three-primer assay consisting of one primer common to the two mating-type idiomorphs (CbMAT-R2; Fig. 7), and two primers specific to each of the mating-types (CbMAT1-F and CbMAT2-R); and 2) a four-primer assay consisting of two primers specific to *MAT1-1-1* (CbMAT1-F and CbMAT1-R) and a primer specific to *MAT1-2-1* (CbMAT2-F2), which was paired with a primer developed by Bolton *et al*.^28^ to amplify a product specific to MAT1-2 isolates.

The PCR mix for both the three-primer and four-primer assays (15 µl total volume) contained 1× PCR buffer (New England Biolabs, Inc., Ipswich, MA) including 1.5 mM MgCl_2_, 0.1 mM dNTPs (New England Biolabs), 0.1 μM of each primer, 0.8 U Taq polymerase (New England Biolabs), and 10 ng of template DNA. PCR conditions included an initial denaturation for 5 min at 95 °C; followed by 34 cycles of denaturation at 95 °C for 30 s; annealing at 62 °C and 58 °C for the three-primer and four-primer assays, respectively, for 30 s; and extension at 68 °C for 30 s; and a final extension at 68 °C for 5 min.

### Tests for random mating

For each field, significant departures of mating-type frequencies from a 1:1 ratio were tested with a chi-square (χ_2_) goodness-of-fit test, for both clone-corrected and non-clone-corrected datasets. To investigate random association of microsatellite loci, the index of association (*I*_*a*_)^94^ and standardized index of association ($\overline{{r}_{d}}$)^78^, which is independent of the number of loci, were estimated after 1,000 permutations in *poppr*, for both clone-corrected and non-clone corrected datasets from each field. Since population structure/admixture can also result in detection of linkage disequilibrium even when the population is randomly mating^20^, tests of random mating were also estimated for the clusters detected by Bayesian analysis, before and after clone-correction. These analyses could not be conducted for the six clusters detected in DAPC analysis due to the small number of the individuals in each cluster (especially after clone correction), which limits the power of LD analysis^20, 95^.

### Detecting recombination

To investigate evidence for recombination in *C. beticola* populations, the proportion of compatible pairs of loci (PrCP)^96^ was estimated using MultiLocus. Methods based on the principle of compatibility among sites/loci are among the most powerful to detect recombination^97^. Two loci are considered compatible (PrCP = 1), if it is possible to account for all the observed genotypes by mutations only; without having to infer homoplasy (reversals, parallelisms, or convergences) or recombination. Under the assumption that recurrent, parallel and reverse mutations are very rare, phylogenetic incompatibility (PrCP < 1) provides evidence of genetic exchange, and may be interpreted as presence of recombination^54^. PrCP was estimated for *C. beticola* populations in each field as well as the clusters detected by Bayesian analysis. The statistical significance for the PrCP test was inferred by comparing the number of compatible pairs of loci in the observed data set to those from a randomly recombined data set in MultiLocus. The null hypothesis of random mating was rejected if more compatible loci than expected in a randomised population were observed (*P* < 0.05)^98, 99^.

## Electronic supplementary material

Supplementary information
