# Supplementary material for: Genetic structure of Cercospora beticola populations on Beta vulgaris in New York and Hawaii
Source: Sci Rep. 2017 May 11;7:1726. doi: 10.1038/s41598-017-01929-4 (PMC5431814; doi:10.1038/s41598-017-01929-4)
Supplement: Supplementary file 1 — Supplementary information [file 41598_2017_1929_MOESM1_ESM.pdf]

Supplementary information

Genetic structure of *Cercospora beticola* populations on *Beta vulgaris* in New York and Hawaii

Niloofar Vaghefi[1], Scot C. Nelson[2], Julie R. Kikkert[3], and Sarah J. Pethybridge[1*]

[1] School of Integrative Plant Science, Plant Pathology & Plant-Microbe Biology Section, Cornell University, Geneva, NY, USA

[2] College of Tropical Agriculture and Human Resources, Department of Tropical Plant and Soil Sciences, University of Hawaii at Manoa, Honolulu, HI 96822, USA

[3] Cornell Cooperative Extension, Canandaigua, NY, USA

* sjp277@cornell.edu

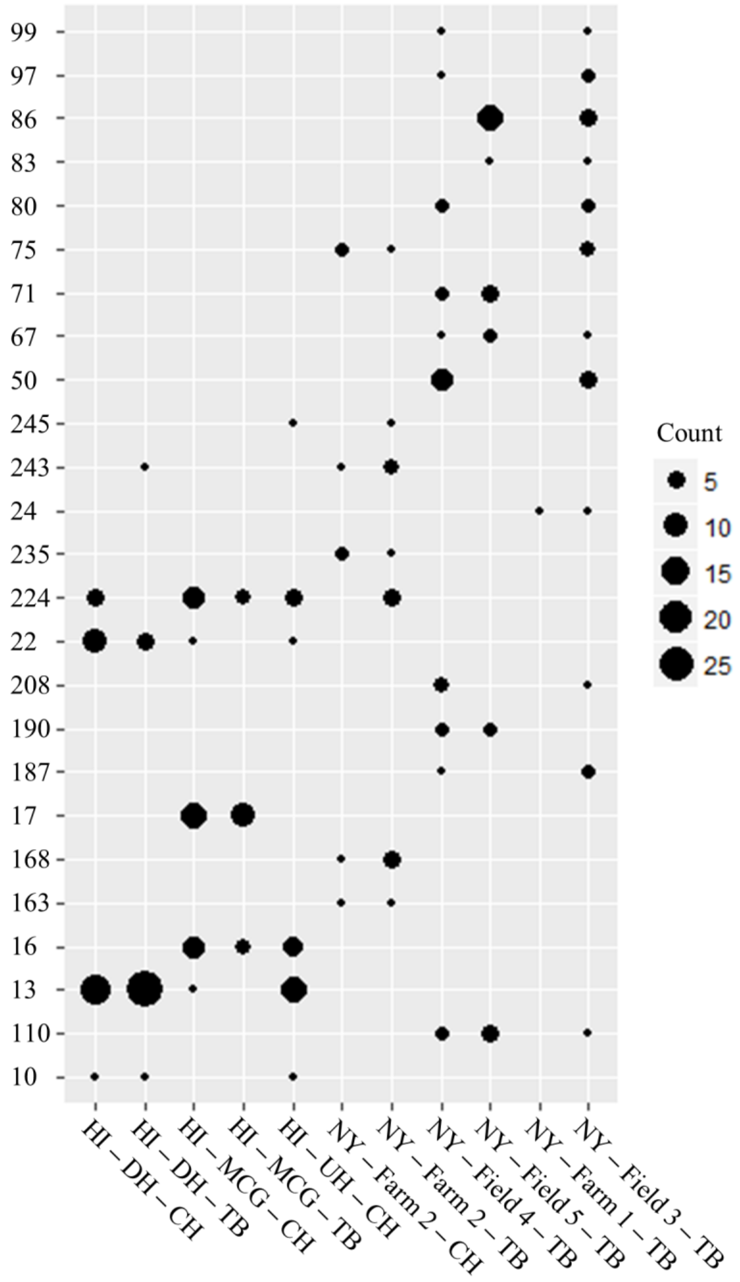

Supplementary Figure 1. Recurrent *Cercospora beticola* multi-locus genotypes (MLGs) that were shared among populations from New York and Hawaii in 2015. Vertical axis shows the MLG number and horizontal axis indicates *C. beticola* populations represented by state – field – host (TB = table beet and CH = Swiss chard).

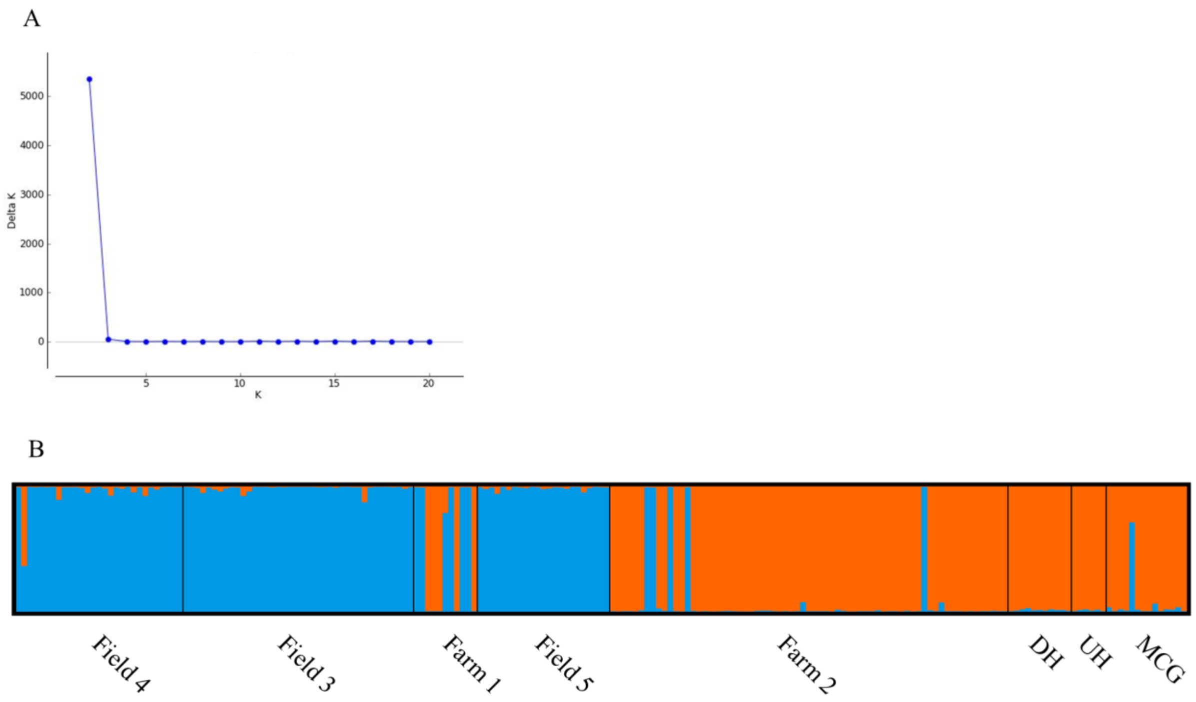

Supplementary Figure 2. Bayesian clustering of *Cercospora beticola* populations collected from New York (Farms 1 and 2, and Fields 3, 4, and 5) and Hawaii (DH, UH, MCG), in 2015. (A) Estimation of ΔK to determine the optimal number of clusters (K); (B) Assignment of *C. beticola* isolates to clusters (blue and orange) based on 10 replicated runs. Each bar represents one isolate and the bar height represents estimated membership fraction of each individual in each of the inferred clusters.

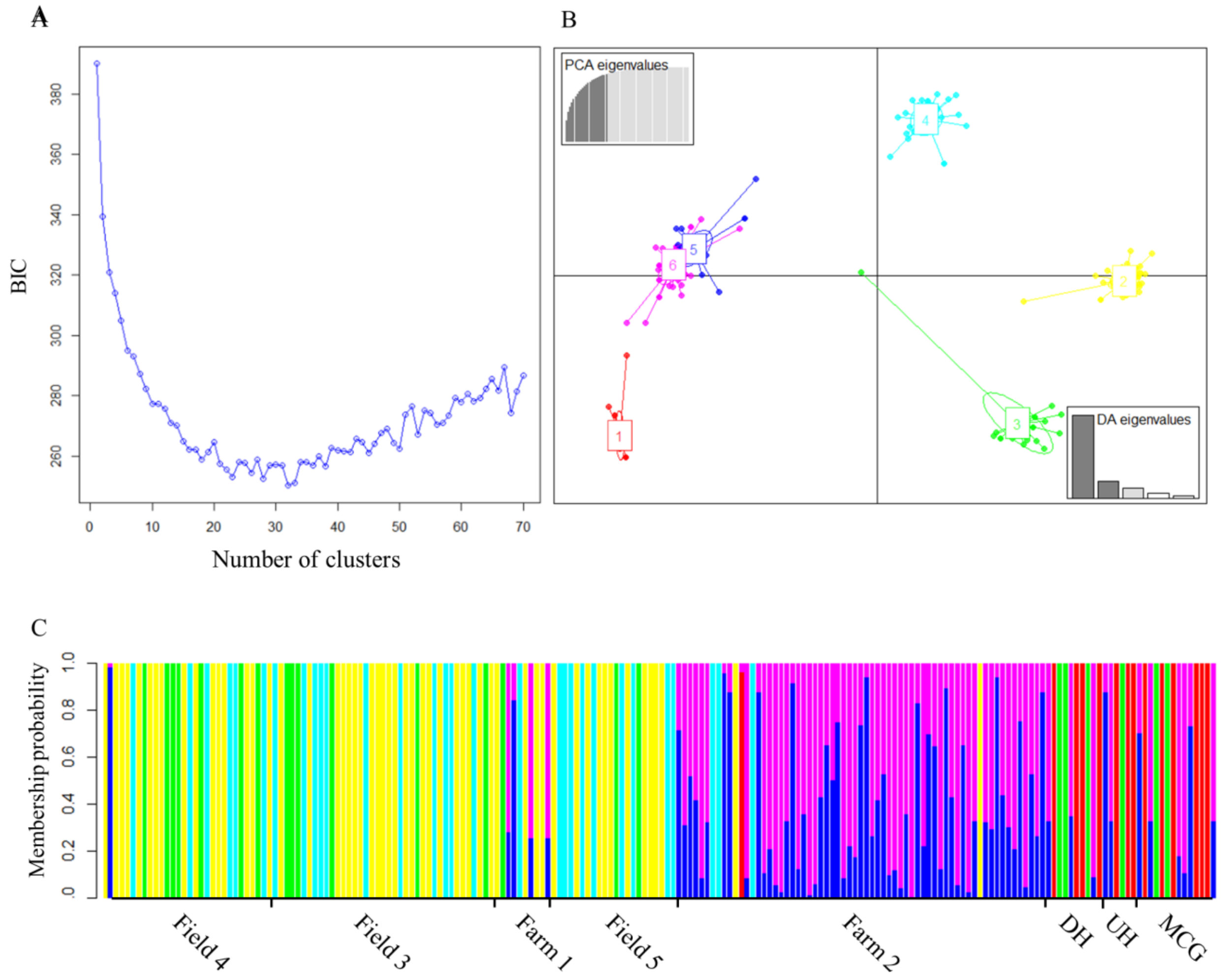

Supplementary Figure 3. Discriminant Analysis of Principal Components (DAPC) for *Cercospora beticola* isolates from New York and Hawaii, 2015; (A) Bayesian Information Criterions (BIC) estimated for different numbers of clusters; (B) Scatter plot of six genetically related clusters; (C) Membership probability of *C. beticola* isolates to the six genetic clusters. Each bar indicates an individual and the colours represent individuals' membership in the predicted clusters. Presence of more than one colour in a bar indicates admixture.
